# Anomaly detection of fermi surface morphology in Co2MnGaxGe1-x via interpretable machine learning

**DOI:** 10.1038/s41598-026-39115-0

**Published:** 2026-04-27

**Authors:** Daichi Ishikawa, Kentaro Fuku, Yoshio Miura, Yasuhiko Igarashi, Yuma Iwasaki, Yuya Sakuraba, Koichiro Yaji, Alexandre Lira Foggiatto, Takahiro Yamazaki, Naoka Nagamura, Masato Kotsugi

**Affiliations:** 1 Department of Material Science and Technology, Tokyo University of Science, Tokyo, Japan; 2 Department of Chemistry, Graduate School of Science, Nagoya University, Aichi, Japan; 3 Faculty of Electrical Engineering and Electronics, Kyoto Institute of Technology, Kyoto, Japan; 4 Research Center for Magnetic and Spintronic Materials (CMSM), National Institute for Materials Science (NIMS), Tsukuba, Japan; 5 Faculty of Engineering, Information and Systems, University of Tsukuba, Ibaraki, Japan; 6 Center for Basic Research on Materials (CBRM), National Institute for Materials Science (NIMS), Ibaraki, Japan; 7 Unprecedented-scale Data Analytics Center (UDAC), Tohoku University, Miyagi, Japan; 8 Research Institute of Electrical Communication (RIEC), Tohoku University, Miyagi, Japan

**Keywords:** Materials science, Spintronics

## Abstract

The Fermi surface provides indispensable insights into the electronic structure of materials. Here, we present a robust analysis framework employing an interpretable machine learning approach to investigate Fermi surfaces. Complex Fermi surface images of the Heusler alloy Co_2_MnGa_x_Ge_1−x_, along with corresponding spin polarization, were analyzed using simple principal component analysis (PCA). Our results reveal that pronounced “jumps” in the PCA space correlate strongly with extrema and inflection points in the spin polarization. Notably, compositions near Ga = 0.94–0.95 exhibit significant changes attributable to the emergence of nodal lines. And the position of nodal lines in momentum space were automatically detected by differential analysis of outlier. Robustness evaluations demonstrate that our method remains effective even under conditions of increased image broadening and noise, mimicking ARPES experimental data. This method can contribute to the analysis of large-scale datasets by detecting non-systematic outliers.

**Supplementary Information:**

The online version contains supplementary material available at 10.1038/s41598-026-39115-0.

## Introduction

The Fermi surface serves as critical information for determining the electronic properties of materials. Its morphology, which evolves in response to crystal structure, elemental composition, and band dispersion, underpins a range of functional properties such as carrier density, magnetic behavior and spin polarization. In spintronic and topological materials^1,2^, exotic features—including Weyl points and nodal lines—emerge on the Fermi surface^3–6^, giving rise to phenomena such as anomalous Nernst effects. Similarly, materials like graphene and borophene exhibit unique electronic states (e.g., Dirac cones and Dirac nodal lines) that facilitate pure spin currents and efficient charge transport^7–10^. Thus, the intricate link between the microscopic structure of the Fermi surface and macroscopic electrical properties has spurred extensive research across diverse material systems^11,12^.

Experimentally, the analysis of Fermi surfaces and band dispersions has relied on angle-resolved photoemission spectroscopy (ARPES). Recent technological advances have significantly improved both angular and energy resolutions, enabling the precise observation of Fermi surface features and band structures^13–15^. The incorporation of spin filters has further allowed for spin-resolved measurements, while next-generation synchrotron facilities are paving the way for high-throughput data acquisition^16^.

Despite these advancements, the interpretation of Fermi surface data remains a labor-intensive process that demands deep expertise. Quantifying the complex shapes of Fermi surfaces often relies on subjective judgment, particularly when selecting regions of interest and elucidating the mechanisms underlying functional properties. Furthermore, noisy band-dispersion data impedes the accurate evaluation of material properties and constitutes a bottleneck in high-throughput analyses. To advance such analyses, machine-learning approaches have been developed for band-dispersion image reconstruction^17,18^, noise reduction^19–21^, and real-space domain mapping^22,23^; however, studies that explicitly focus on the topology of the Fermi surface remain scarce^24^. As discussed above, Fermi surface topology underpins a variety of functional properties. Therefore, developing methods capable of efficiently screening large collections of experimental spectra to identify Fermi surfaces with distinctive morphologies is essential.

In this situation, dimensionality reduction techniques interpretable within the framework of unsupervised learning are effective^25,26^. Although the fine structure of a Fermi surface can exhibit intricate variations, the limited diversity in elemental composition and structural configurations renders the data inherently sparse, with a low effective dimensionality. This characteristic makes dimensionality reduction a particularly effective tool for our analysis. Furthermore, as future applications are likely to focus on novel materials and unexplored physical phenomena—scenarios where labeled data is scarce—unsupervised machine learning offers a practical and powerful approach.

Among various methods, principal component analysis (PCA) stands out as a simple yet highly interpretable unsupervised technique^27^. PCA compresses complex variations into a low-dimensional space based on Euclidean metrics, thereby allowing abrupt changes in the dataset to emerge as outliers. The abrupt modulation of the Fermi surface is typically associated with a marked change in material properties, enabling the detection of outliers by utilizing distances within PCA space. This approach, well-established in the field of outlier detection, also enables us to assess the robustness of our methodology by analyzing inter-data point distances in PCA scatter plots.

In this study, we focus on the Co₂MnGaₓGe₁₋ₓ (CMGG) Heusler alloy, a prototypical spintronic material, and examine whether composition-dependent modulations of the Fermi surface and related physical properties can be analyzed using principal component analysis (PCA) and distance-based anomaly detection. CMGG has been widely investigated both theoretically and experimentally because of its characteristic electronic structure, including half-metallicity and nodal-line features. Owing to its anomalous Nernst effect, arising from nodal lines on the Fermi surface, and its high spin polarization, CMGG provides an ideal model system for this work^28–32^.

**Fig. 1:**
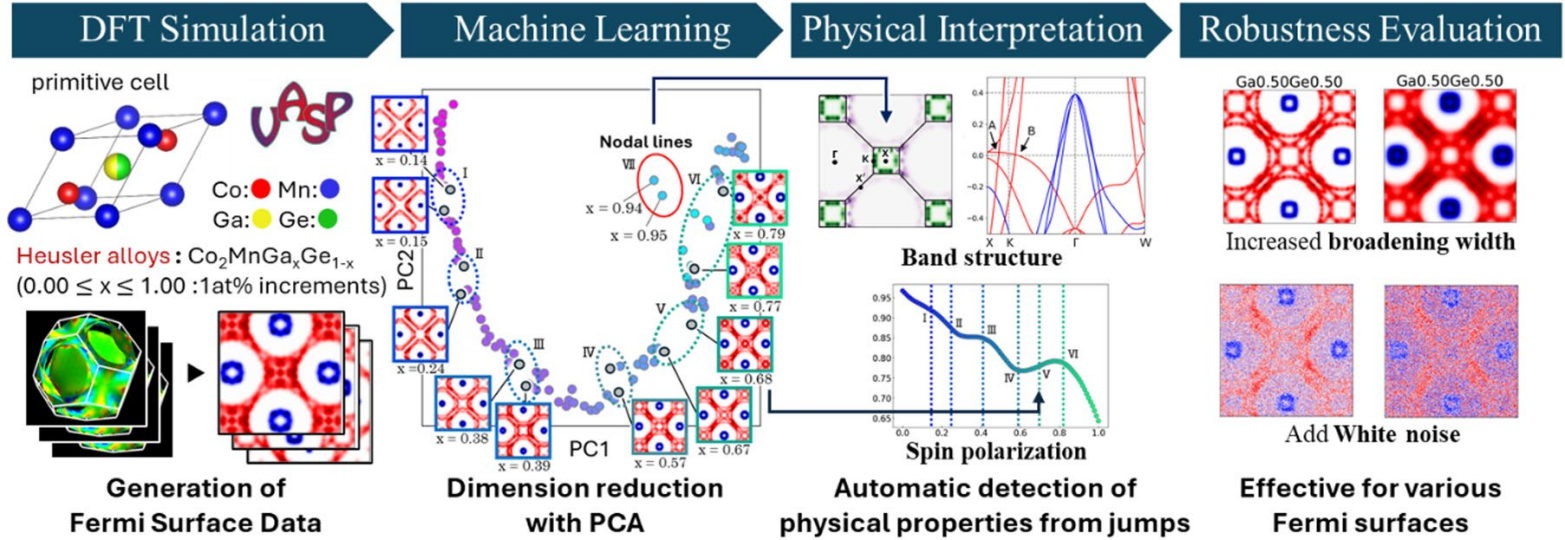
Workflow for Fermi Surface Analysis Using Machine Learning.

The workflow is summarized in Figs. 1 and S1. Using first-principles calculations, we generated a comprehensive band-structure dataset that captures the compositional dependence of the Fermi surface. From these band structures, we extracted the *k*x–*k*y plane through the Γ point and applied a blurring procedure to obtain image data that roughly approximate ARPES measurements. We then employed PCA to analyze the evolution of the data and to identify outliers in the reduced space. By examining the compositions flagged as characteristic, we show that they coincide with regimes associated with the emergence of nodal lines and with extrema in the spin-polarization ratio, i.e., compositions that are directly relevant to physical properties. Finally, we evaluated the robustness of the method under adverse conditions—such as high noise and reduced resolution—to assess its applicability to experimental data acquired under less-than-ideal ARPES conditions^33^.

Initially, density functional theory (DFT) simulations are conducted to generate Fermi surface images of the Heusler alloy with varying compositions. Subsequently, PCA is applied to these images to visualize the data in a reduced, low-dimensional space. The PCA results are then compared with band structures and spin polarization values to elucidate the contribution of electronic states. Finally, the noise tolerance is evaluated to establish a robust analytical framework.

## Methods

### Band structure calculations

The electronic band structure of the Heusler alloy Co₂MnGaₓGe_1−x_ (CMGG) was computed using density functional theory (DFT) as implemented in the Vienna Ab initio Simulation Package (VASP)^34^. Calculations were performed within the semi-local generalized gradient approximation (GGA-PBE), employing a 20 × 20 × 20 Monkhorst-Pack k-point mesh. In constructing the pseudopotentials, the valence states for Co (3d, 4s), Mn (3p, 3d, 4s), and Ga/Ge (3d, 4s, 4p) were explicitly considered. Spin degree of freedom was incorporated, and both the lattice structure and magnetic moments were allowed to relax. Because Ga and Ge are neighboring elements and the number of d-electrons is unchanged throughout the Ga–Ge substitution, we adopted the virtual crystal approximation (VCA)^35^ as the alloying model in this study. By varying the composition in 1 atomic percent increments, we generated a total of 101 distinct datasets.

### Fermi surface image generation

The Fermi surfaces were visualized using FermiSurfer^36^ by extracting the coordinates corresponding to the Fermi surface on the *k*ₓ–*k*_y_ plane that includes the Γ point. These coordinates were then converted into 255 × 255 pixel grayscale images by convolving them with a Gaussian distribution, as specified in Eq. (1). The resulting images were normalized through cropping so that the luminance values were scaled to lie within the range of 0 to 1.

1 $$\:\begin{array}{c}d=\sqrt{{\left(x-{x}_{\mathrm{c}\mathrm{e}\mathrm{n}\mathrm{t}\mathrm{e}\mathrm{r}}\right)}^{2}+{\left(y-{y}_{\mathrm{c}\mathrm{e}\mathrm{n}\mathrm{t}\mathrm{e}\mathrm{r}}\right)}^{2}},\:gradient=\mathrm{exp}\left(\frac{-{d}^{2}}{2{\sigma\:}^{2}}\right)\end{array}$$

### Spin polarization

For the analysis, spin polarization was chosen as the key physical quantity. It was computed from the majority and minority spin densities of states at the Fermi energy, D_↑_(*E*_F_) and D_↓_(*E*_F_), as obtained from the first-principles calculations using the expression provided below. This metric enabled a quantitative evaluation of the magnetic characteristics inherent to the system.

2 $$\:\begin{array}{c}P=\frac{{D}_{\uparrow\:}\left({E}_{F}\right)-{D}_{\downarrow\:}\left({E}_{F}\right)}{{D}_{\uparrow\:}\left({E}_{F}\right)+{D}_{\downarrow\:}\left({E}_{F}\right)}\end{array}$$

### Dimensionality reduction via PCA

The high-dimensional Fermi surface image data were first flattened into one-dimensional vectors, after which principal component analysis (PCA) was applied for dimensionality reduction to assess inter-data similarities and variations. PCA was selected for its parameter-free, linear projection properties that facilitate straightforward interpretation^37^. In this study, each 255 × 255 grayscale image—representing pixel luminance—was reshaped into a one-dimensional vector and then concatenated with a corresponding vector in which the majority spin values were multiplied by + 1 and the minority spin values by − 1. This procedure yielded a single input vector of length 130,050 for each dataset, thereby constructing a data space in which similarities and outliers could be effectively identified and correlated with variations in spin polarization.

### Robustness evaluation

The robustness of the proposed methodology was investigated with respect to increased Fermi surface broadening and the introduction of noise. The broadening parameter σ in Eq. (1) was varied from 4 to 6 to simulate an enhanced broadening effect, and white noise (with a peak signal-to-noise ratio of 4.76 dB)^38^ was added to the Fermi surface images. Subsequent PCA analyses were conducted under these altered conditions to evaluate the stability and reliability of the approach in the presence of measurement imperfections.

## Results discussion

### Machine learning analysis of fermi surface morphology and spin polarization

**Fig. 2:**
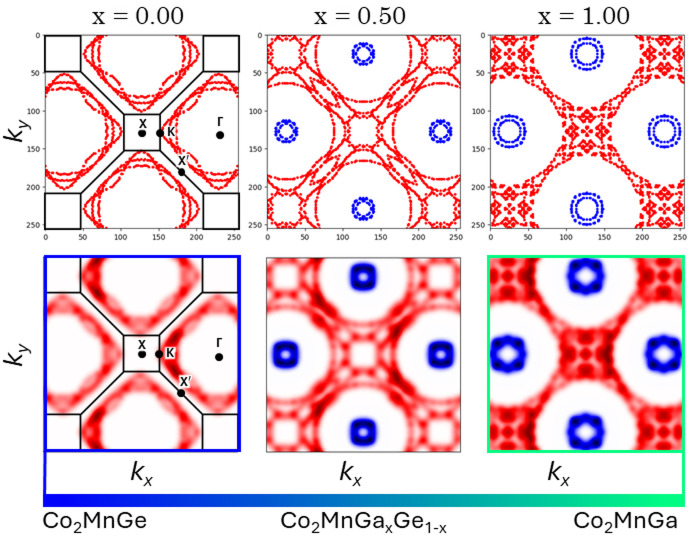
Fermi Surface Images Obtained from First-Principles Calculations.

The upper panel displays the plot data exported from FermiSurfer, while the lower panel shows the corresponding blurred Fermi surface images mimicking ARPES experimental data.

Figure 2 presents representative Fermi surface images obtained from our DFT calculations. In these images, the up-spin bands are rendered in red while the down-spin bands appear in blue; dashed lines delineate the Brillouin zone boundaries. These features are consistent with the ARPES images reported by Takashi Kono et al.^39^, thereby validating our computational approach (Figure S2). Notably, for Co₂MnGe (i.e., at X = 0), only the red up-spin Fermi surface is observed, resulting in a high spin polarization. In contrast, as the Ga content increases, additional blue down-spin features emerge at the fourfold Γ points. The expanding down-spin contributions near the Γ point lead to an overall reduction in spin polarization, while the morphology of the up-spin bands near the X and K points also varies with composition, yielding a complex evolution of the spin polarization. To quantitatively interpret these nontrivial changes, we employed principal component analysis (PCA) for machine learning on the Fermi surface images.

**Fig. 3:**
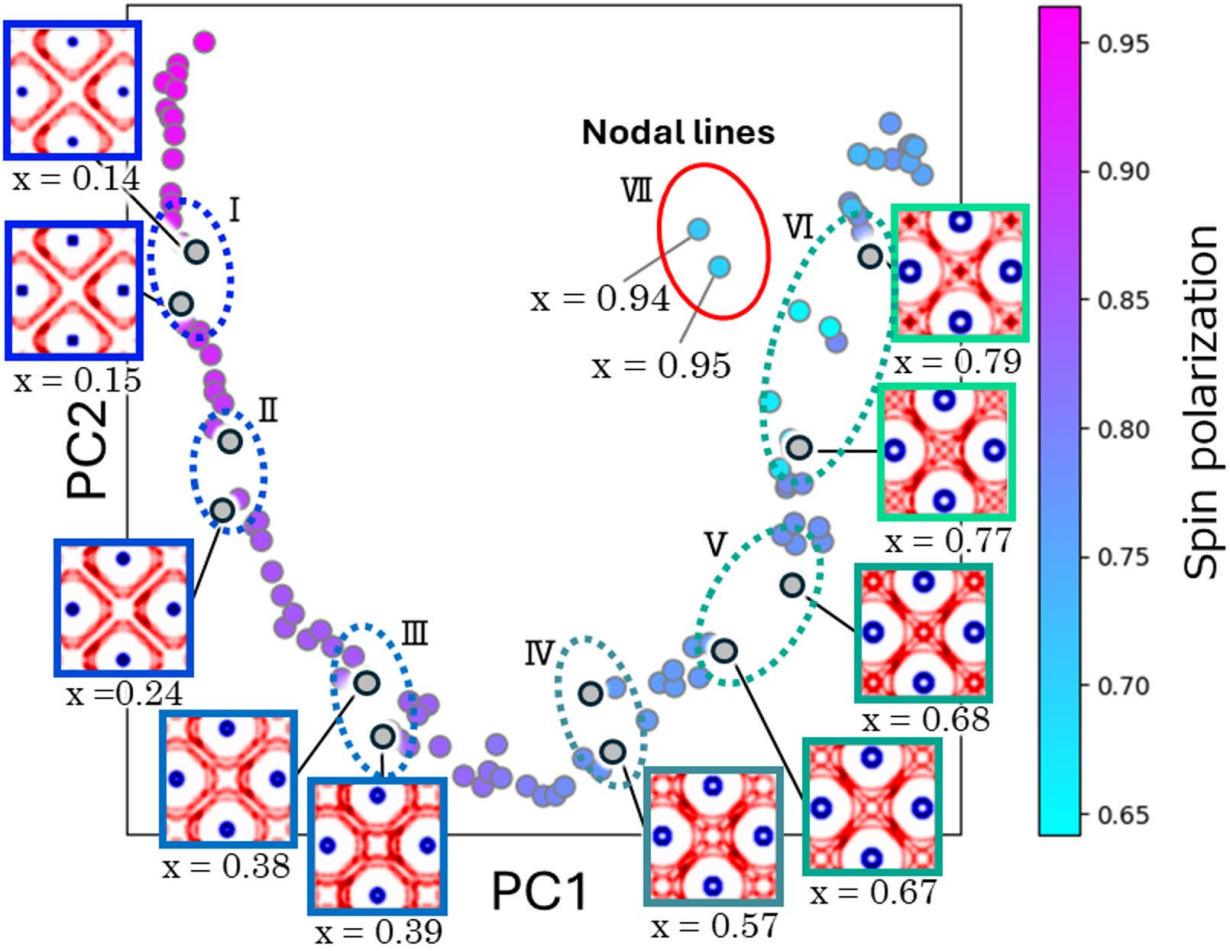
Dimensionality Reduction of Fermi Surfaces via PCA.

The two-dimensional PCA mapping is shown, where the horizontal axis represents PC1 and the vertical axis represents PC2. Each plotted point corresponds to compositions, and the color indicates spin polarization. Representative Fermi surface images are displayed around the plot. There are several “jumps” in a continuous trend. I-VI jumps correspond to extrema and inflection points in the spin polarization, and largest VII jumps occurring in the compositions where the nodal lines appear on the Fermi surface.

PCA was applied to the Fermi surface image dataset, and the data were projected onto a two-dimensional space, as shown in Fig. 3 and Figure S3. Each point in the plot corresponds to the Fermi surface data of a specific composition. The first principal component (PC1) and the second principal component (PC2) account for 64.7% and 16.1% of the total variance, respectively, with a cumulative contribution of 80.8% when combined (Figure S4). This high retention of information in two dimensions underscores the effectiveness of the dimensionality reduction. Moreover, PC1 shows a strong correlation with the Ga/Ge composition (correlation coefficient of 0.966), suggesting that it robustly captures the compositional influence on the Fermi energy of CMGG (Figure S5, S6).

**Fig. 4:**
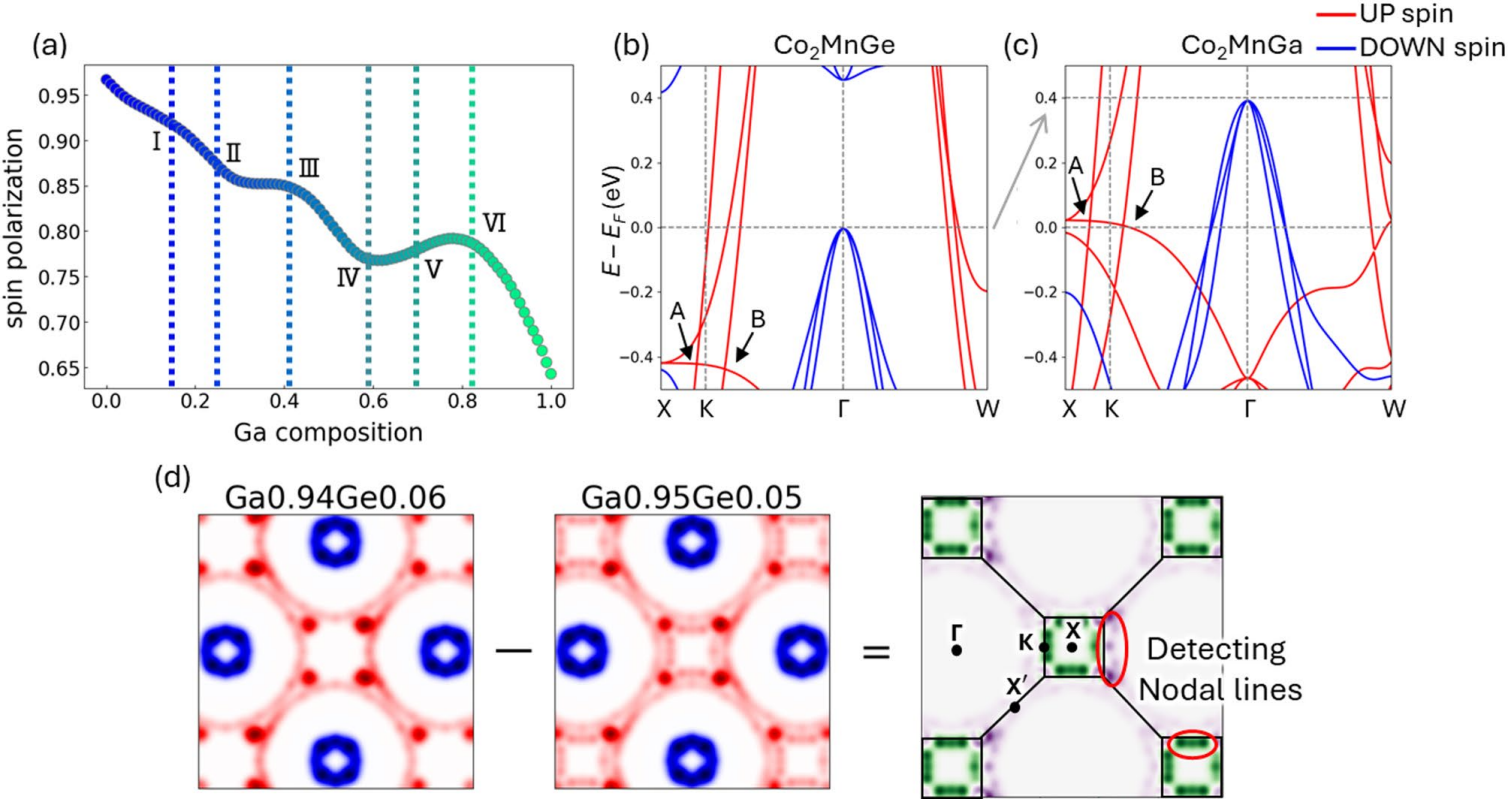
Correlation Between Spin Polarization and Electronic Structure.

(a) Variation in spin polarization for Co₂MnGaₓGe_1−x_ is presented, with the horizontal axis indicating the compositional ratio and the vertical axis representing the spin polarization values. Vertical line indicate the “jumps” of PCA mapping, which is accord to extrema and inflection point. (b, c) The calculated band dispersion of Co₂MnGe and Co₂MnGa is shown, with markers A and B denoting nodal lines. (d) Fermi surface images of the compositions (Ga = 0.94 and 0.95) which show largest “jumps” and their differential images are presented. Highlighted regions of differential images are consistent to the nodal lines.

In the PC2 direction, several pronounced “jumps” are observed (Figure S5, S6). Since the Euclidean distance between points in the PCA space reflects the similarity of the input images, large jumps indicate significant changes in Fermi surface morphology. In Fig. 3, these jumps are labeled I through VI. The compositions corresponding to these jumps match the extrema or inflection points in the spin polarization curve shown in Fig. 4(a). For example, jump I at compositions Ga = 0.14 and 0.15 corresponds to marked morphological changes near the Γ point and aligns with a maximum in spin polarization. Similarly, jump II at Ga = 0.23 and 0.24 corresponds to changes between the Γ and W points, matching a minimum in the spin polarization. Jump V, observed at Ga = 0.67 and 0.68, is associated with the emergence of a band near the X point, which in turn corresponds to an inflection point in the spin polarization. Jumps III through VI similarly coincide with other extrema in spin polarization. These findings indicate that the PCA projection of the Fermi surface morphology provides an effective visual index for tracking variations in spin polarization.

Furthermore, a pronounced deviation is evident at Ga compositions between 0.94 and 0.95 (labeled as outlier VII in Fig. 3, highlighted by a red circle). This deviation corresponds to the appearance of nodal lines, as reported by Kazuki Sumida et al..^28^ In Co₂MnGa, nodal lines have been observed to emerge slightly above the Fermi energy^40^, leading to anomalous Hall and Nernst effects^29,41–43^. Figures 4(b) and (c) display the band structures along the X–K–Γ–W path for Co_2_MnGa_x_Ge_1−x_ with x = 0 and 1.0. These results indicate that compositional changes in CMGG are largely consistent with a rigid band model^44–46^, wherein shifts in the Fermi energy are observed. The points labeled A and B in Figs. 4(b) and (c) correspond to the crossing points between the nodal lines and the high-symmetry Γ-K-X line, with the composition corresponding to outlier VII marking the regime where these nodal lines approach the Fermi level. Figure 4(d) further illustrates the differential Fermi surface images for the extracted compositions; the dark green and purple regions coincide with nodal line A (appearing between the K and X points) and nodal line B (emerging near the K point in the Γ–K segment), which also align with areas of enhanced Berry curvature reported in previous studies.

We interpret these correspondences as follows. Because CMGG is well described within a rigid-band model, the band filling changes continuously with composition (electron count), and PC1 primarily captures the resulting monotonic expansion or contraction of the Fermi surface associated with each band. In contrast, PC2 correlates with changes in Fermi-surface morphology, and its large excursions—“jumps”—can be regarded as signaling pronounced, non-systematic changes of the Fermi surface. The spin polarization is, via Eq. (2), given by the difference in the density of states at the Fermi level, to which the Fermi-surface morphology contributes. Moreover, because PC2 provides a robust representation of the spin-polarization behavior (as discussed below), we interpret switches in spin-polarization trends associated with changes in Fermi-surface morphology as being detected as large jumps along PC2. Similarly, the emergence of a nodal line, which is accompanied by a band crossing and thus a substantial change in Fermi-surface morphology, can manifest as a pronounced jump in the PCA space.

In addition, we compared segmentation based on the “jump” identified by this method with conventional clustering techniques (k-means on a PCA plane). Clustering labels were assigned using known labels: change points (maxima, minima, inflection points) in the spin polarization and the composition at which nodal lines appear. The confusion matrices were then compared. Both methods exhibit regions around cluster 5 that are difficult to partition. However, the partition based on “jumps” successfully isolates the cluster where nodal lines appear. (Figure S7) From these results, it can be said that the proposed method successfully highlighted data (in this paper, composition) exhibiting significant changes in Fermi-surface morphology. Detailed analysis of the composition of interest revealed that the composition is related to the spin polarization changes, the emergence of nodal lines (and their position in momentum space). Therefore, this method demonstrates the ability to extract compositions associated with distinct (anomalous) changes in Fermi-surface morphology that correlate with specific variations in physical properties. This suggests potential applicability to a range of systems, such as strongly correlated materials with flat bands and Weyl/Dirac semimetals with multiple nodal features. At the same time, the core of the approach is the detection of non-systematic anomalies superimposed on systematic trends. In practice, one must carefully assess whether a given material system and dataset satisfy this premise before applying the method.

### Robustness evaluation

Experimental ARPES data are often compromised by factors such as limited light source monochromaticity, the performance of electron analyzers, and temperature-induced broadening, which collectively blur the Fermi surface features. Additionally, short measurement times or low absorption cross-sections can lead to significant noise due to low photoelectron counts, thereby degrading the signal-to-noise ratio. Although the simulated maps are only a coarse approximation to full ARPES images, we assessed the applicability of our method under such challenging conditions by evaluating its robustness against broadening and noise additions. Our approach maps the Fermi surface data onto a two-dimensional PCA space and identifies “jumps” — instances where the Euclidean distance between adjacent compositions exceeds a defined threshold. This threshold was established by constructing a histogram of distances between neighboring data points; values in the top 10% of the distribution were classified as significant jumps.

**Fig. 5:**
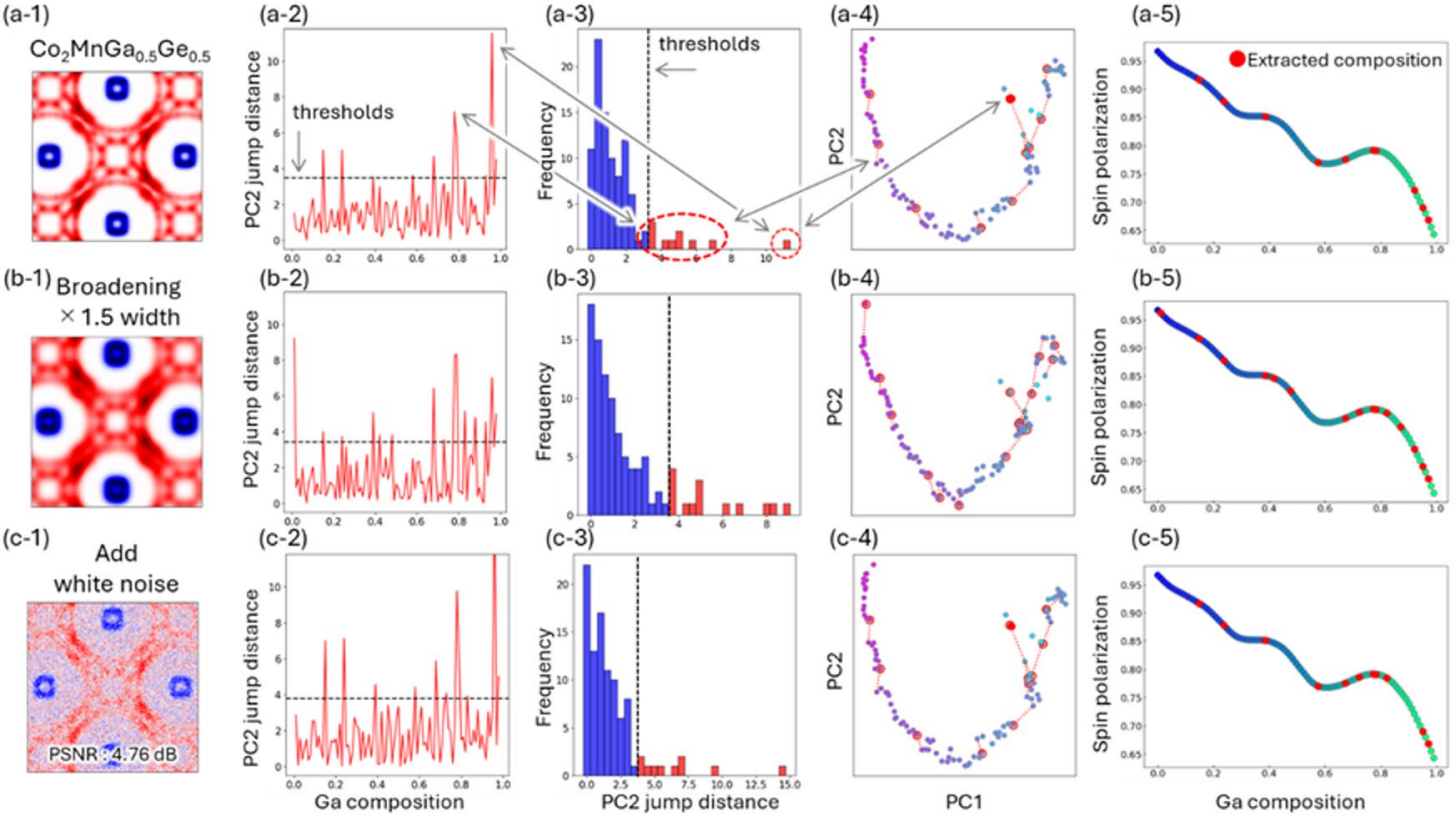
Quantitative Analysis of Fermi Surface Data Under Varying Conditions.

(1) Representative examples of Fermi surface images subjected to different conditions of broadening and noise. (2, 3) Plots of PC2 differences for each composition and their corresponding histograms. (4, 5) PCA mapping and spin polarization plots with compositions exhibiting the top 10% of PC2 differences highlighted. (a) Quantitative analysis results based on the raw Fermi surface data are presented. (b) Analysis results obtained when the broadening width is increased by a factor of 1.5 are shown, demonstrating the method’s performance under degraded resolution. (c) Analysis outcomes with added white noise are provided, confirming the robustness of the PCA-based approach even under high-noise conditions.

Figure 5(a) shows the results of the quantification of “jumps” in the Fermi surface image in the previous section, and Fig. 5(a-1) is representative example of the Fermi surface images. Figure 5(a-2) plots the absolute differences along the PC2 direction as a function of CMGG composition, while Fig. 5(a-3) presents a corresponding histogram with the top 10% of values highlighted in red. Figure 5(a-4) overlays these identified compositions on the PCA map, using red outlines and dashed lines to indicate the critical points. The composition with the largest difference is marked by a red circle and corresponds to the regime where nodal lines appear. Additionally, Fig. 5(a-5) emphasizes that the top 1% of the jumps automatically extract the composition associated with the nodal lines, while the top 10% capture compositions where the spin polarization exhibits marked changes.

Regarding the influence of broadening, our analysis shows that with proper threshold adjustments, similar levels of performance can be achieved (Figure S8, S9). Figure 5(b) demonstrates that even when the Fermi surface images are intentionally blurred to mimic experimental broadening, the data from the Ga-rich side become more pronounced while the extraction of changes on the Ge-rich side becomes more challenging. However, by adjusting the threshold to capture the upper 15% of the distance distribution, we are still able to extract the critical compositions. Even when the broadening width is increased by a factor of 1.5, slight threshold modifications enable the identification of compositions essential for understanding spin polarization. These results suggest that our method remains effective even under conditions of poor momentum resolution or when the bands are not sharply defined due to long fluorescence lifetimes.

When strong white noise (with a PSNR of 4.76 dB) was added to the Fermi surface images, the robustness of the method was further confirmed. As illustrated in Fig. 5(c), although the cumulative variance captured by the PCA decreased in the presence of noise, our approach still successfully identified the compositions associated with variations in spin polarization and the emergence of nodal lines. Our experiments indicate that noise levels exceeding a PSNR of 4.76 dB present significant challenges for analysis (Figure S10). Nonetheless, for materials with low photon absorption cross-sections or in scenarios where measurement time is limited, resulting in low S/N ratios, our method offers a viable pathway for effective analysis.

In summary, these results demonstrate that the PCA-based approach is robust against both broadening and noise effects, and it holds significant promise for facilitating high-throughput ARPES measurements and the automated analysis of complex electronic structures in materials.

## Conclusion

In this study, with a view toward the analysis of large-scale ARPES datasets, we focused on unsupervised machine-learning techniques and examined the effectiveness of the simplest PCA approach using simulated Fermi-surface images of the CMGG Heusler alloy. The analysis revealed that pronounced modulations appearing as “jumps” in the PCA space correspond to extrema and inflection points in the spin-polarization curve. Outliers were observed at the compositions x = 0.94 and 0.95 in the Ga content, which we attribute to the emergence of a nodal line in the vicinity of the Fermi level. The position of the nodal line in momentum space was detected by a differential analysis of the Fermi surface. A quantitative evaluation of the jump distances showed that the top 1% of compositions (in terms of jump magnitude) correspond to the appearance of the nodal line, while the top 10% have a particularly strong impact on spin polarization. These findings suggest that PCA can be used to extract compositions associated with characteristic changes in physical properties.

Looking ahead, incorporating energy dispersion into the present framework could be explored to probe correlations between band-structure modulations and physical properties. Because the method is formulated as a human-in-the-loop analysis that ranks samples based on inter-sample distances in data space, it may in principle also be used to flag candidates associated with unknown properties inferred from unusual Fermi-surface morphologies.

On the other hand, the present approach is specifically designed to detect non-systematic anomalies superimposed on an underlying systematic trend; therefore, care must be taken to ensure that the target material system and dataset satisfy this premise before the method is applied. In this context, the scheme could be useful for organizing and screening data from high-throughput, sequential ARPES mapping on composition-gradient (combinatorial) films, and thus for supporting emerging workflows that combine combinatorial deposition with high-throughput ARPES. More generally, given its robustness in the simulated tests performed here, the framework may offer a complementary aid for exploring large-scale experimental ARPES datasets when used together with appropriate domain expertise.

## Supplementary Information

Below is the link to the electronic supplementary material.

Supplementary Material 1

## Data Availability

The authors declare that the data supporting the conclusions of this study can be accessed in the article, its supplementary materials, and on Github, with further information obtainable from the corresponding authors on request.

## References

[1] Fu, L., Kane, C. L. & Mele, E. J. Topological insulators in three dimensions. *Phys. Rev. Lett.* **98**, 106803 (2007). 17358555 10.1103/PhysRevLett.98.106803

[2] Pesin, D. & MacDonald, A. H. Spintronics and pseudospintronics in graphene and topological insulators. *Nat. Mater.* **11**, 409–416 (2012). 22522641 10.1038/nmat3305

[3] Sumida, K. et al. Prolonged duration of nonequilibrated Dirac fermions in neutral topological insulators. *Sci. Rep.* **7**, 14080 (2017). 29074864 10.1038/s41598-017-14308-w PMC5658381

[4] Hsieh, D. et al. A topological Dirac insulator in a quantum spin hall phase. *Nature* **452**, 970–974 (2008). 18432240 10.1038/nature06843

[5] Yan, B. & Felser, C. Topological materials: Weyl semimetals. *Annual Rev. Condens. Matter Phys.* **8**, 337–354 (2025).

[6] Soluyanov, A. A. et al. Type-II Weyl semimetals. *Nature* **527**, 495–498 (2015). 26607545 10.1038/nature15768

[7] Geim, A. K. & Novoselov, K. S. The rise of graphene. *Nat. Mater.* **6**, 183–191 (2007). 17330084 10.1038/nmat1849

[8] Castro Neto, A. H., Guinea, F., Peres, N. M. R., Novoselov, K. S. & Geim, A. K. The electronic properties of graphene. *Rev. Mod. Phys.* **81**, 109–162 (2009).

[9] Sugawara, K. et al. Selective fabrication of free-standing ABA and ABC trilayer graphene with/without Dirac-cone energy bands. *NPG Asia Mater.* **10**, e466 (2018).

[10] Fu, B. B. et al. Dirac nodal surfaces and nodal lines in ZrSiS. *Sci. Adv.* **5**, eaau6459 (2019). 31058219 10.1126/sciadv.aau6459 PMC6499591

[11] Iwasaki, T. et al. Bulk and surface electronic structures of CePdX (X = As, Sb) studied by 3d-4f resonance photoemission. *Phys. Rev. B*. **61**, 4621–4628 (2000).

[12] Sekiyama, A. et al. Itinerant bulk 4f character of strongly valence-fluctuating CeRu_2_ observed by high-resolution Ce 3d-4f resonance photoemission. *Solid State Commun.* **121**, 561–564 (2001).

[13] Johnson, P. D. Spin-polarized photoemission. *Rep. Prog Phys.* **60**, 1217–1304 (1997).

[14] Yaji, K. et al. High-resolution three-dimensional spin- and angle-resolved photoelectron spectrometer using vacuum ultraviolet laser light. *Rev. Sci. Instrum.* **87**, 053111 (2016). 27250396 10.1063/1.4948738

[15] Yaji, K. & Tsuda, S. Visualization of spin-polarized electronic States by imaging-type spin-resolved photoemission microscopy. *Sci. Technol. Adv. Materials: Methods*. **4**, 2328206 (2024).

[16] Yuan, J., Stanev, V., Gao, C., Takeuchi, I. & Jin, K. Recent advances in high- throughput superconductivity research. *Supercond Sci. Technol.* **32**, 123001 (2019).

[17] Ekahana, S. A. et al. Transfer learning application of self-supervised learning in ARPES. *Mach. Learn. Sci. Technol.* **4**, 035021 (2023).

[18] Xian, R. P. et al. A machine learning route between band mapping and band structure. *Nat. Comput. Sci.* **3**, 101–114 (2022). 38177954 10.1038/s43588-022-00382-2 PMC10766556

[19] Kim, Y. et al. Deep learning-based statistical noise reduction for multidimensional spectral data. *Rev. Sci. Instrum.* **92**, 073901 (2021). 34340442 10.1063/5.0054920

[20] Iwasawa, H. et al. Efficiency improvement of spin-resolved ARPES experiments using Gaussian process regression. *Sci Rep* **14,** 20970 (2024). 10.1038/s41598-024-66704-8 PMC11420225 39313521

[21] Restrepo, F., Zhao, J. & Chatterjee, U. Denoising and feature extraction in photoemission spectra with variational auto-encoder neural networks. *Rev. Sci. Instrum.* **93**, 065106 (2022). 35778019 10.1063/5.0090051

[22] Imamura, M. & Takahashi, K. Unsupervised learning of spatially-resolved ARPES spectra for epitaxially grown graphene via non-negative matrix factorization. *Sci. Rep.* **14**, 24200 (2024). 39406827 10.1038/s41598-024-73795-w PMC11480436

[23] Peng, H. et al. Super resolution convolutional neural network for feature extraction in spectroscopic data. *Rev. Sci. Instrum.* **91**, 033905 (2020). 32259998 10.1063/1.5132586

[24] Iwasawa, H. et al. Quantitative measure of correlation strength among intertwined many-body interactions. *Phys. Rev. Res.* **5**, 043266 (2023).

[25] Suzuki, Y., Hino, H., Kotsugi, M. & Ono, K. Automated Estimation of materials parameter from X-ray absorption and electron energy-loss spectra with similarity measures. *NPJ Comput. Mater.* **5**, 39 (2019).

[26] Kunii, S., Masuzawa, K., Fogiatto, A. L., Mitsumata, C. & Kotsugi, M. Causal analysis and visualization of magnetization reversal using feature extended Landau free energy. *Sci. Rep.* **12**, 19892 (2022). 36446857 10.1038/s41598-022-21971-1 PMC9709087

[27] Abdi, H. & Williams, L. J. Principal component analysis. *Wiley Interdiscip Rev. Comput. Stat.* **2**, 433–459 (2010).

[28] Sumida, K. et al. Spin-polarized Weyl cones and giant anomalous Nernst effect in ferromagnetic heusler films. *Commun. Mater.* **1**, 89 (2020).

[29] Guin, S. N. et al. Anomalous Nernst effect beyond the magnetization scaling relation in the ferromagnetic heusler compound Co_2_MnGa. *NPG Asia Mater.* **11**, 16 (2019).

[30] De Groot, R. A., Mueller, F. M., Van Engen, P. G. & Buschow, K. H. J. New class of materials: Half-Metallic ferromagnets. *Phys. Rev. Lett.* **50**, 25 (1983).

[31] Kubler, J., Williams, A. R. & Sommers, C. B. Fortnation and coupling of magnetic moments in heusler alloys. *Phys. Rev. B*. **28**, 1745–1755 (1983).

[32] Galanakis, I., Dederichs, P. H. & Papanikolaou, N. Slater-Pauling behavior and origin of the half-metallicity of the full-Heusler alloys. *Phys. Rev. B Condens. Matter Mater. Phys.* **66**, 1–9 (2002).

[33] Iwasawa, H. et al. Development of laser-based scanning µ-ARPES system with ultimate energy and momentum resolutions. *Ultramicroscopy* **182**, 85–91 (2017). 28666139 10.1016/j.ultramic.2017.06.016

[34] Kresse, G. & Furthmü, J. Efficient iterative schemes for Ab initio total-energy calculations using a plane-wave basis set. *Phys. Rev. B*. **54**, 11169–11186 (1996). 10.1103/physrevb.54.11169 9984901

[35] Eckhardt, C., Hummer, K. & Kresse, G. Indirect-to-direct gap transition in strained and unstrained Sn_x_Ge_1–x_ alloys. *Phys. Rev. B Condens. Matter Mater. Phys.* **89**, 165201 (2014).

[36] Kawamura, M. & FermiSurfer Fermi-surface viewer providing multiple representation schemes. *Comput. Phys. Commun.* **239**, 197–203 (2018).

[37] Jolliffe, I. T. & Cadima, J. Principal component analysis: a review and recent developments. *Philosophical Trans. Royal Soc. A: Math. Phys. Eng. Sci.* **374**, 20150202 (2016). 10.1098/rsta.2015.0202 PMC4792409 26953178

[38] Hore, A. & Ziou, D. Image Quality Metrics: PSNR vs. SSIM. In *2010 20th International Conference on Pattern Recognition,* 2366–2369 (IEEE, 2010).

[39] Kono, T. et al. Visualizing Half-Metallic bulk band structure with multiple Weyl cones of the heusler ferromagnet. *Phys. Rev. Lett.* **125**, 216403 (2020). 33274987 10.1103/PhysRevLett.125.216403

[40] Kono, T. et al. Three-dimensional bulk fermi surfaces and Weyl crossings of Co_2_MnGa thin films underneath a protection layer. *Phys. Rev. B*. **104**, 195112 (2021).

[41] Xiao, D., Yao, Y., Fang, Z. & Niu, Q. Berry-phase effect in anomalous thermoelectric transport. *Phys. Rev. Lett.* **97**, 026603 (2006). 16907470 10.1103/PhysRevLett.97.026603

[42] Xiao, D., Chang, M. C. & Niu, Q. Berry phase effects on electronic properties. *Rev. Mod. Phys.* **82**, 1959–2007 (2010).

[43] Sakai, A. et al. Giant anomalous Nernst effect and quantum-critical scaling in a ferromagnetic semimetal. *Nat. Phys.* **14**, 1119–1124 (2018).

[44] Balke, B. et al. Properties of the quaternary half-metal-type heusler alloy Co_2_Mn_1–x_ Fe_x_ Si. *Phys. Rev. B Condens. Matter Mater. Phys.* **74**, 104405 (2006).

[45] Varaprasad, B. S. D. C. S. et al. Spin polarization and Gilbert damping of Co_2_Fe(Ga _x_Ge _1–x_) heusler alloys. *Acta Mater.* **60**, 6257–6265 (2012).

[46] Sakuraba, Y. et al. Evidence of fermi level control in a half-metallic heusler compound Co_2_MnSi by Al-doping: comparison of measurements with first-principles calculations. *Phys. Rev. B*. **81**, 144422 (2010).

