# Supplementary material for: Anomaly detection of fermi surface morphology in Co2MnGaxGe1-x via interpretable machine learning
Source: Sci Rep. 2026 Apr 27;16:12698. doi: 10.1038/s41598-026-39115-0 (PMC13121614; doi:10.1038/s41598-026-39115-0)
Supplement: Supplementary file 1 — Supplementary Material 1 [file 41598_2026_39115_MOESM1_ESM.docx]

Supporting Information:

Anomaly Detection of Fermi Surface Morphology in Co_2_MnGa_x_Ge_1-x_ via Interpretable Machine Learning

Daichi Ishikawa^1^, Kentaro Fuku^1,2,*^, Yoshio Miura^3,4^, Yasuhiko Igarashi^5^, Yuma Iwasaki^6^, Yuya Sakuraba^4^, Koichiro Yaji^6,7^, Alexandre Lira Foggiatto^1^, Takahiro Yamazaki^1^, Naoka Nagamura^1,6,8^, Masato Kotsugi^1^

^1^Department of Material Science and Technology, Tokyo University of Science, Tokyo, Japan

^2^Department of Chemistry, Graduate School of Science, Nagoya University, Aichi, Japan;

^3^Faculty of Electrical Engineering and Electronics, Kyoto Institute of Technology, Kyoto, Japan

^4^Research Center for Magnetic and Spintronic Materials (CMSM), National Institute for Materials Science (NIMS), Tsukuba, Japan

^5^Faculty of Engineering, Information and Systems, University of Tsukuba, Ibaraki, Japan

^6^Center for Basic Research on Materials (CBRM), National Institute for Materials Science (NIMS), Ibaraki, Japan

^7^Unprecedented-scale Data Analytics Center (UDAC), Tohoku University, Miyagi, Japan

^8^Research Institute of Electrical Communication (RIEC), Tohoku University, Miyagi, Japan

**Data preparation**


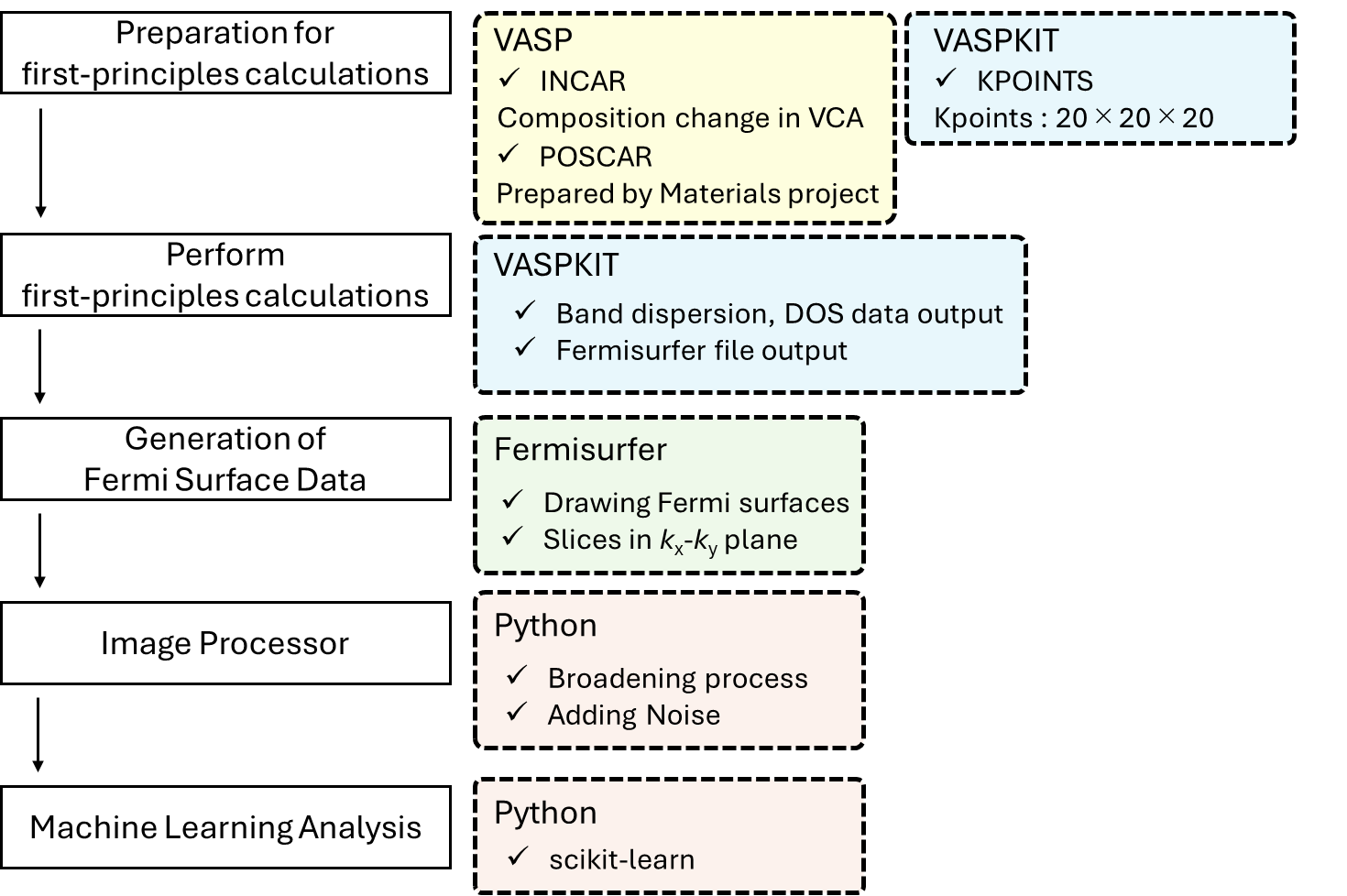


**Fig. S1 Workflow diagram from first-principles calculations to PCA analysis.** After the first-principles calculations, band dispersion and DOS data were output using VASPKIT, and FRMSF files were output for Fermisurfer, which outputs coordinate data sliced in the *k*_x_-*k*_y_ plane and preprocesses Fermi surface images using Python. Machine learning was performed using scikit-learn.


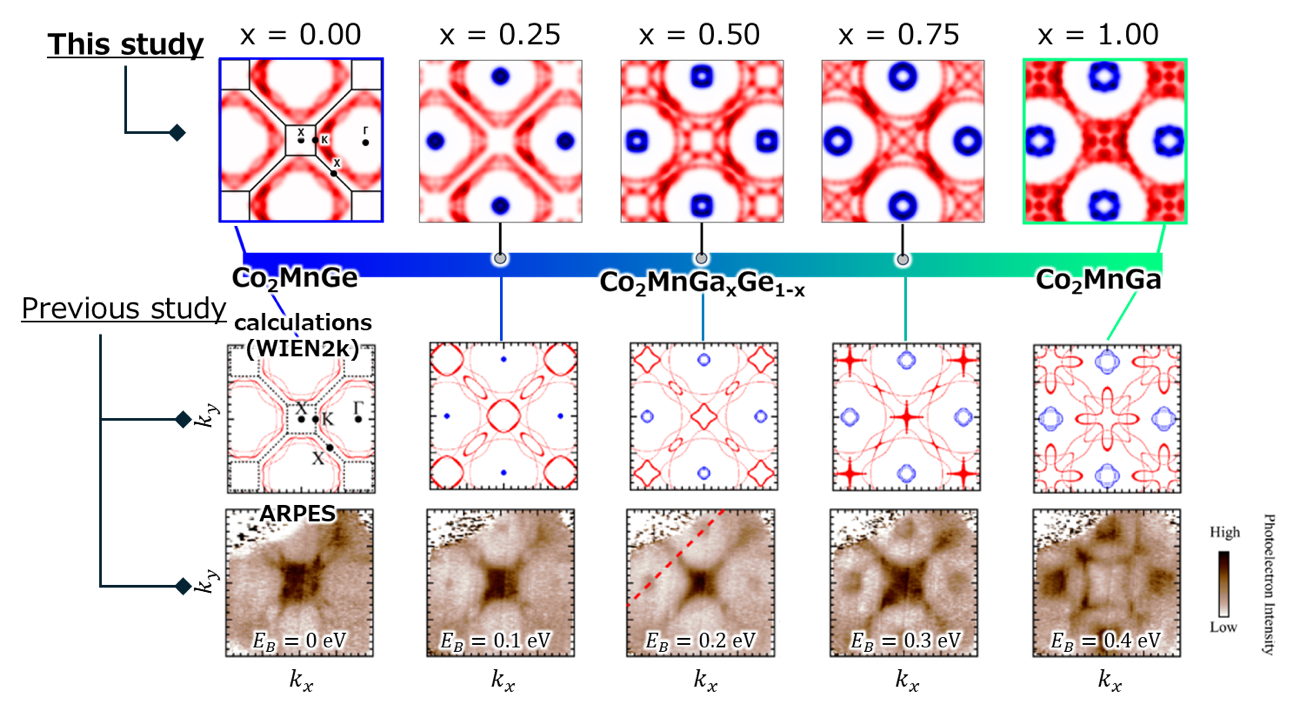


**Fig. S2 Simulated images corresponding to experimental ARPES images from the prior study.** This figure adapted with permission from Ref. [39]. Copyrighted by the American Physical Society.

**The results of Principal Component Analysis (PCA)**


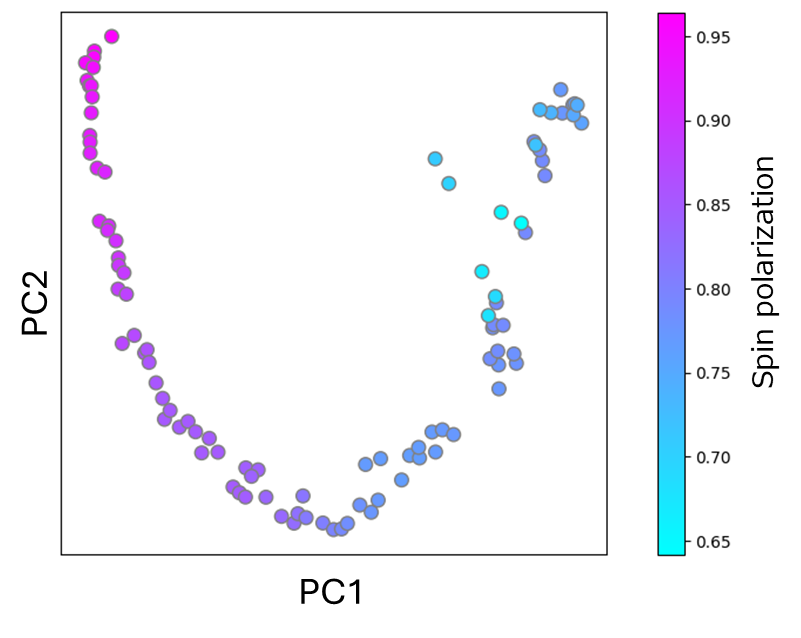


**Fig. S3 PC1 vs. PC2 plots of Fermi surfaces analysis.** Colors represent values of spin polarization.


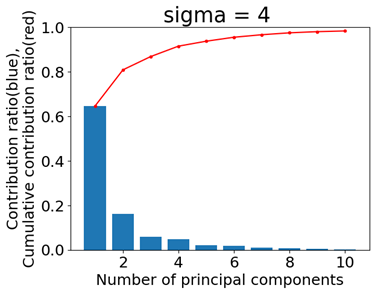


**Fig. S4 PCA contribution rates plot (broadening sigma=4).** The histogram shows the contribution rate per principal component, and the red line shows the cumulative contribution rate.


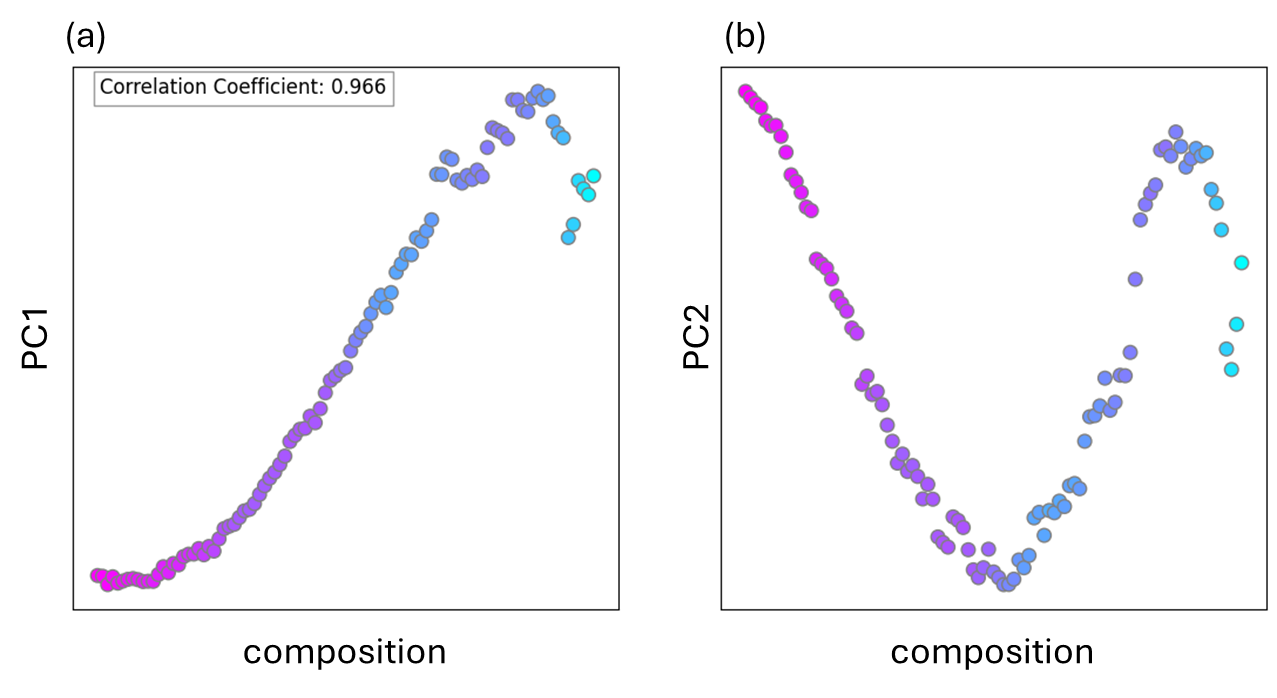


**Fig. S5 Plots of PC1, 2 vs. composition.** (a) PC1 vs. composition. The correlation coefficient is 0.966, indicating that PC1 represents a composition ratio. (b) PC2 vs. composition. The jumps in the data indicate that PC2 is some signature of the shape of the Fermi surface and spin polarization.


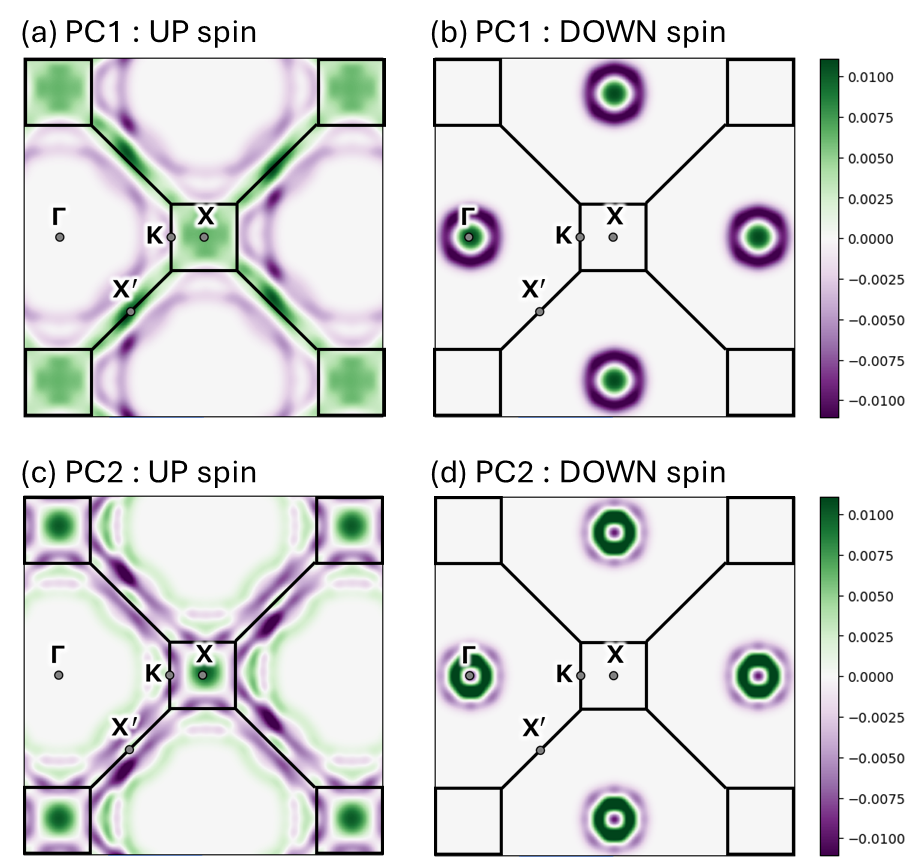


**Fig. S6 Eigenvectors for PC1 and PC2 for each spin.**


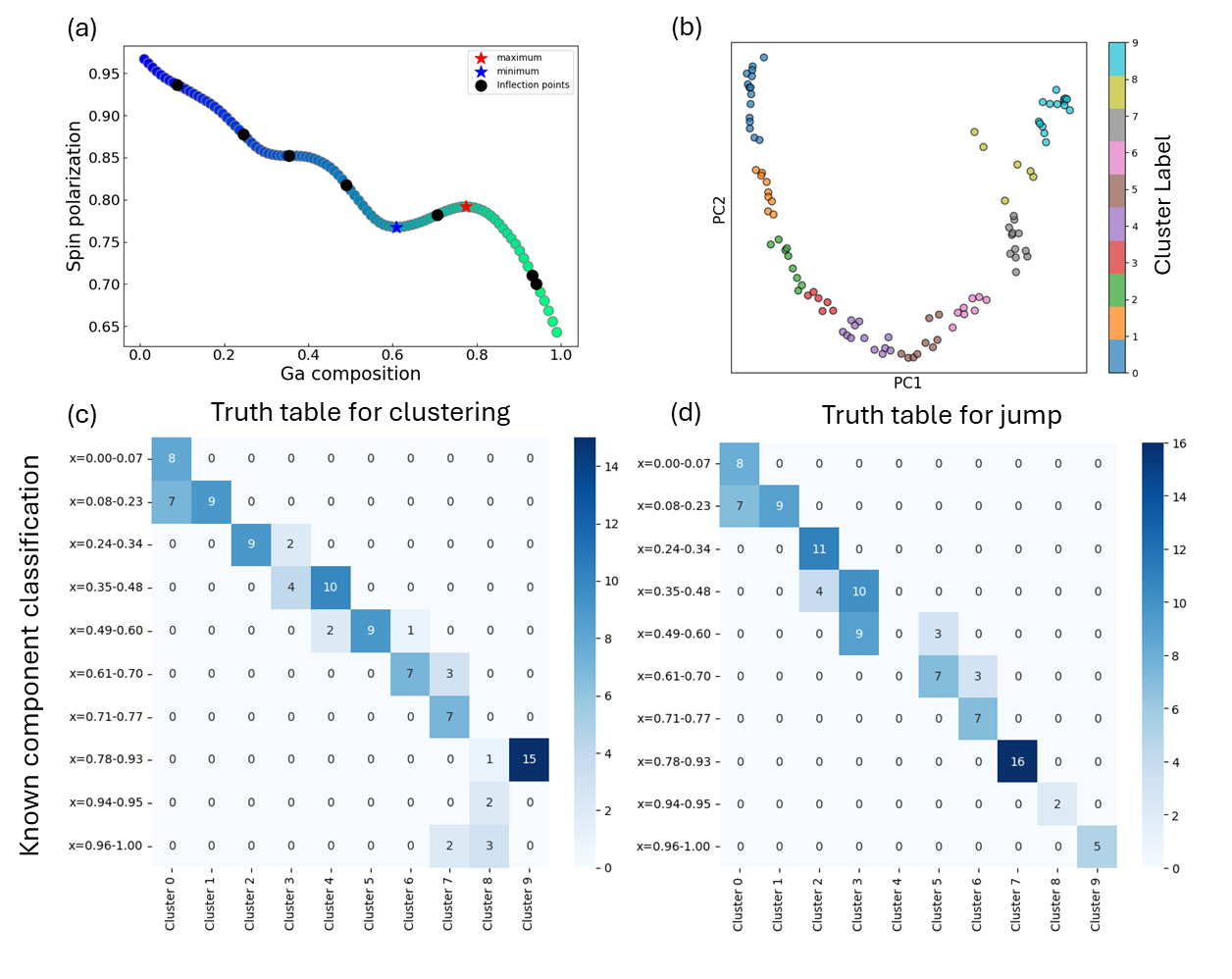


**Fig. S7 Classification comparison.** (a) Salient landmarks in the spin-polarization profile. (b) k-means clustering in the PCA embedding. (c–d) Confusion (contingency) matrices for (c) k-means and (d) PC2-jump–based segmentation, evaluated against regime labels defined by salient spin-polarization landmarks and compositions at which nodal lines appear on the Fermi surface.

**Robustness evaluation for broadening**


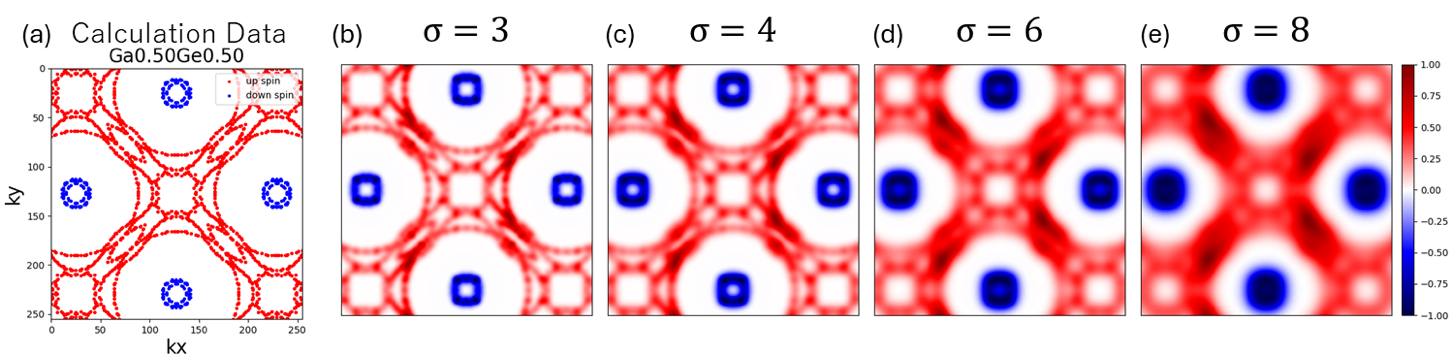


**Fig.S8 The broadened Fermi surface images.** (a)raw calculation data (b, c, d, e) broadened Fermi surface images with sigma=3, 4, 6, 8.


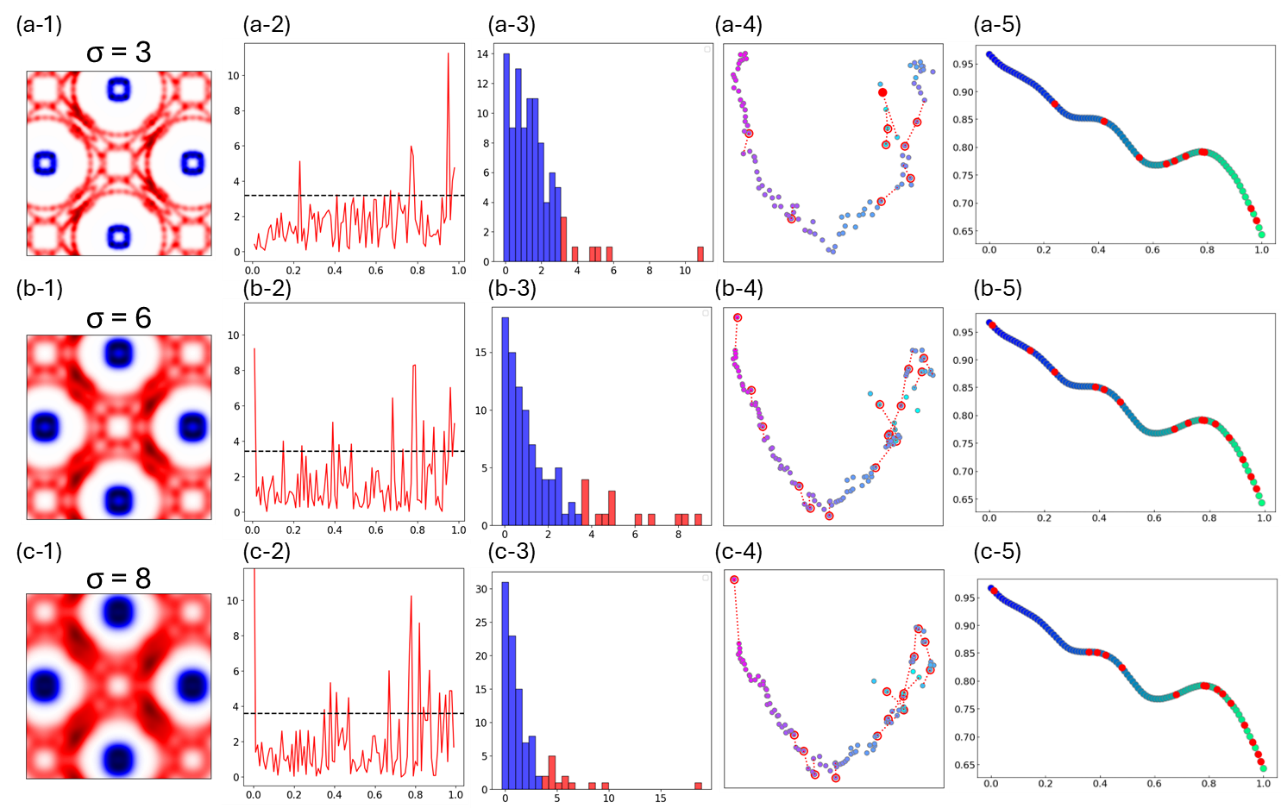


**Fig.S9 The results of Fermi surfaces analysis with broadened images.** (1) Representative examples of Fermi surface images subjected to different conditions of broadening and noise. (2, 3) Plots of PC2 differences for each composition and their corresponding histograms. (4, 5) PCA mapping and spin polarization plots with compositions exhibiting the top 10% of PC2 differences highlighted. (a, b, c) corresponding with sigma=3, 6, 8.

The composition with emergence of nodal lines are extracted in any cases.

**Robustness evaluation of noises**

PSNR (Peak Signal to Noise Ratio): The ratio of maximum power to noise. A large PSNR indicates that the image degradation is small. It is used as an objective evaluation measure.

$$PSNR=10\log_{10} \frac{1}{e^{2}} \left[ dB \right]$$

The maximum luminance value of the Fermi surface is set to 1. The noise power $e^{2}$ is equal to the MSE (Mean Squared Error) of the original and composite images.

$$e^{2}=MSE=\frac{1}{N}\sum_{i} \left( x\left[ i \right]-y\left[ i \right] \right)^{2}$$

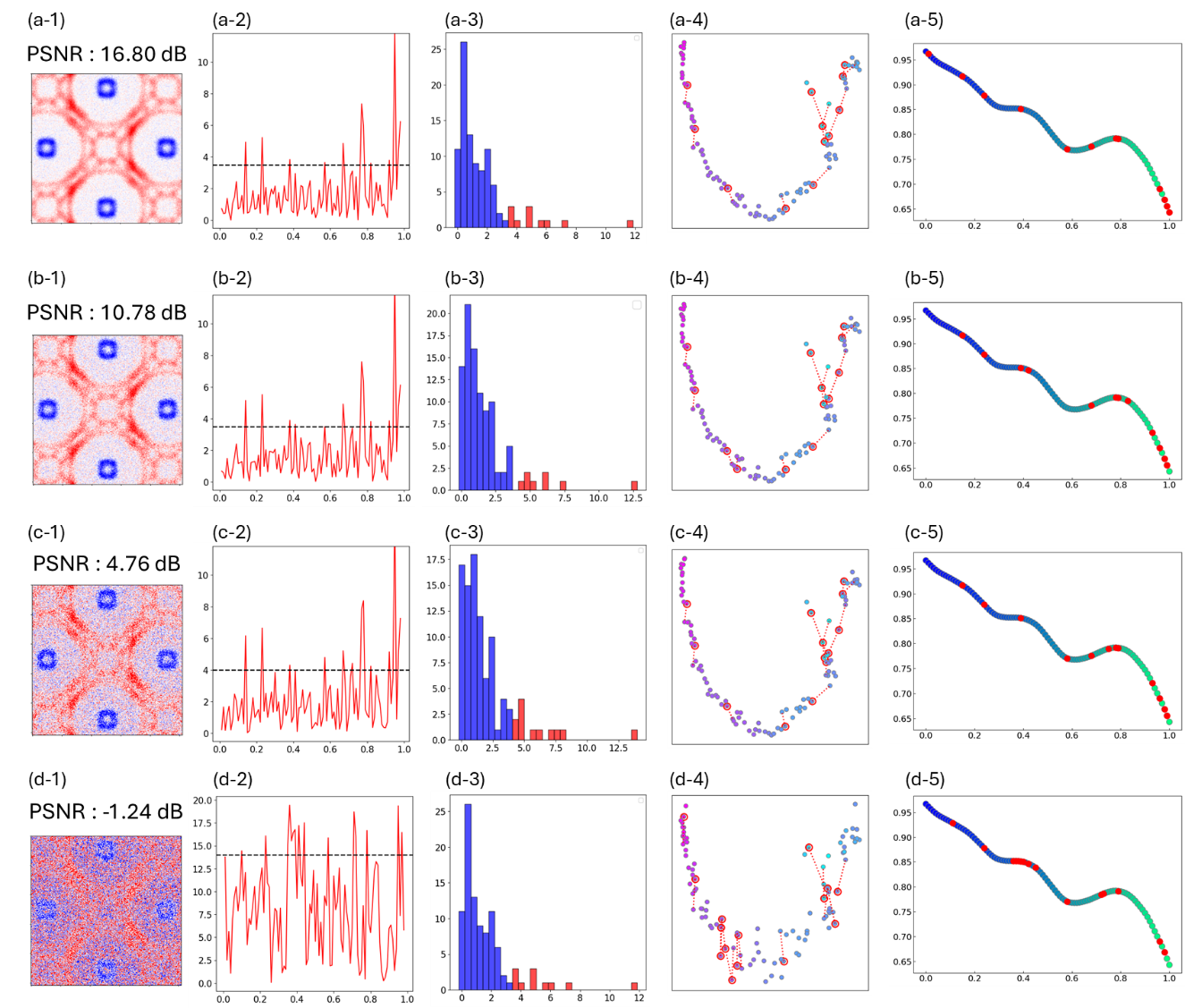


**Fig.S10 The results of Fermi surfaces analysis with noise-added images.** (1-5) indicate same as Fig5. (a-d) corresponding with noise level, PSNR=16.80, 10.78, 4.76, -1.24.
